# Nightmares, Chronotype, Urbanicity, and Personality: An Online Study

**DOI:** 10.3390/clockssleep2030029

**Published:** 2020-09-22

**Authors:** Michael Schredl, Anja S. Göritz

**Affiliations:** 1 Central Institute of Mental Health, Medical Faculty Mannheim/Heidelberg University, 68159 Mannheim, Germany; 2 Psychology Department, University of Freiburg, 79085 Freiburg, Germany; goeritz@psychologie.uni-freiburg.de

**Keywords:** nightmares, nightmare distress, chronotype, urbanicity, personality

## Abstract

Chronotype refers to individual differences in sleep timing (“owls” and “larks”) and “eveningness” has been associated with nightmares. However, it has not been tested as to whether neuroticism mediates this relationship. Urbanicity refers to being raised in an urban region and/or currently living in an urban region and is associated with heightened risk for developing mental disorders, and thus might be related to nightmare frequency and nightmare distress. Overall, 2492 persons (1437 women, 1055 men) completed an online survey between 23 March 2015 and 8 April 2015. The mean age of the sample was 47.75 ± 14.41 years. The findings indicate that the previously reported relationship between chronotype and nightmare frequency was mediated by neuroticism and “morningness” was related to higher dream recall compared to persons with a late bedtime preference. Urbanicity was not related to nightmare frequency but to lower nightmare distress, raising the interesting question as to whether beliefs about nightmares might be an important variable that contributes to nightmare distress. Based on the few studies so far, there are still many unresolved questions about the interaction between nightmares, chronotype, and urbanicity.

## 1. Introduction

Nightmares are defined as extended, extremely dysphoric and well-remembered dreams [1]. Current etiological models of nightmares emphasize the interplay between dispositional factors (affect distress) like neuroticism and/or trait susceptibility and state factors (affect load) like current stress levels [2]. Diagnosing a nightmare disorder (ICD 10: F51.5) requires that nightmares cause clinically significant distress such as, e.g., mood disturbance during the day, sleep resistance (bedtime fear), and/or intrusive nightmare imagery during the day [1]. Thus, it is important to study not only factors associated with nightmare frequency—most studies focused on nightmare frequency correlates [3]—but to study specifically the factors related to nightmare distress. The most obvious factor, of course, is nightmare frequency itself [4,5,6,7,8]. However, it was demonstrated that other factors like neuroticism, female gender, and openness to experiences also contribute independently to nightmare distress (nightmare frequency was statistically controlled in these analyses) [9]. Based on the etiological nightmare models, all factors that are associated with stress, neuroticism, mental disorders, and so on can increase nightmare frequency [2,10,11]. Chronotype refers to individual differences in sleep timing (“owls” and “larks”) and shows high heritability of about 50 in US American citizens [12]. As eveningness (the “owl” chronotype) is associated with higher neuroticism [13,14] and a higher risk for mental disorders [15], the findings that eveningness is related to nightmare frequency [16,17] would fit with the model. These studies, however, did not control for neuroticism and other variables and this seems important as Randler et al. [18] were able to show that the association between nightmare frequency and eveningness was no longer significant if age, gender, neuroticism, and the other four Big Five personality factors were statistically controlled. Unfortunately, this study did not look at a possible contribution of eveningness on nightmare distress.

Urbanicity, i.e., being raised in an urban region and/or currently living in an urban region, is associated with a higher prevalence rate of schizophrenia and mood disorders [19,20]. While two studies [21,22] found a small but significant relationship between the place of residence size and nightmare frequency, this was not corroborated by another study [23]. Another study [24] reported slightly elevated nightmare frequencies in very small towns and very large cities, i.e., a U-shaped relationship between population size and nightmare frequency. Neither of these studies included stress and/or neuroticism measures to control for mediator effects of these variables. Interestingly, dream recall frequency was higher in persons living in big cities compared to persons who live in rural areas [25,26], but this was not replicated by Stepansky et al. [22]. To summarize, the two factors, chronotype and urbanicity, might be related to nightmare frequency but this association might be simply explained by neuroticism/stress scores as mediators.

The aim of the present study was to study the relationship between nightmare frequency, urbanicity, and chronotype while accounting for possible mediating effects of neuroticism and socio-demographic variables like age, gender, and education. In order to replicate previous findings, we also studied the relationship between chronotype, urbanicity, and dream recall frequency.

## 2. Results

The distribution regarding dream recall frequency is depicted in Table 1. More than 50% of the sample recalled their dreams once a week or more often, whereas a minority never recalled dreams. About 9% of the participants stated that they experience nightmares once a week or more often (Table 2), whereas 20% reported never having nightmares. About 5% of the participants who completed the nightmare distress item experience their nightmares as very distressing (Table 3). Low nightmare distress was reported by about 45% of the sample. The means and standard deviations of chronotype and personality variables are depicted in Table 4. The distribution of the self-rated chronotype was as follows: definitely evening type (*n* = 481 [= 19.30%]), more evening than morning type (*n* = 743 [= 29.81%]), more morning than evening type (*n* = 797 [= 31.98%]), and definitely morning type (*n* = 471 [= 18.90%]).

The regression analysis for the four-item chronotype score indicated that morningness is higher in older participants and associated with conscientiousness, whereas openness to experience is associated with eveningness (Table 5). Persons living in larger cities also reported lower chronotype scores (more eveningness). Very small effects were found for education (higher education associated with morningness) and for extraversion (higher scores associated with morningness). No effects were present for neuroticism and agreeableness.

Prior to analyzing the nightmare variables, the effect of chronotype and urbanicity on dream recall was studied—controlling for age, gender, education, and personality factors. The two analyses indicated that dream recall frequency is related to eveningness (small effect) if nightmare frequency was also added into the regression analysis (Analysis 2 in Table 6). Place of residence size (urbanicity) was not related to dream recall frequency. Nightmare frequency was related to age, neuroticism, and openness to experience (Table 7), and to a lesser extent to education and conscientiousness. Although there was a bivariate, a significant association between eveningness and nightmare frequency (r = 0.100, *p* < 0.0001, *n* = 2492; reported in [18]), controlling for age, gender, education, place of residence size, and personality, however, yielded a non-significant finding. A variety of factors contributed to nightmare distress with nightmare frequency as the major factor, followed by neuroticism, female gender, openness to experience, lower education, higher age, and living in smaller towns (Table 7).

## 3. Discussion

The main finding is that chronotype and urbanicity did not contribute independently from other factors like gender, neuroticism, and openness to experience to nightmare frequency. In addition, the previously reported [25,26] positive correlation between dream recall frequency and place of residence size could not be replicated. However, persons living in an urban environment experienced less nightmare distress (small effect) than persons living in rural environments—controlling for personality, nightmare frequency, education, gender, and age.

Several methodological issues have to be addressed. Although the age range and the diversity regarding education of the present sample were large, it was not a representative sample. As participants volunteered to participate in a dream study, nightmare frequency was higher compared to representative samples [21,23] and education levels were higher compared to the German population [27]. Nevertheless, the magnitude of the correlation coefficient between neuroticism and nightmare frequency was comparable to previous studies [9,10,28,29], indicating that the personality correlates of nightmares might not have been biased in a marked way due to sampling issues. Regarding the chronotype, we found the typical increase in morningness with age [30] and the correlation with conscientiousness [13,14], supporting the validity of the present findings. In our sample, morning types were as frequent as evening types, whereas in the original German MEQ sample [31], the evening types were more frequent than morning types. However, this difference is very likely to be explained by the differences in age means as the Griefahn sample included almost exclusively individuals below 30 years of age, whereas the mean age of the present sample is about 48 years. Moreover, the finding that evening types are slightly more frequent in urban settings compared to rural settings was also in line with previous studies [32,33]. In addition, the reliability of the abbreviated chronotype questionnaire (four items) was sufficiently high.

Nightmare frequency was measured retrospectively, and several researchers [34,35,36] have argued that retrospective measures underestimate the prevalence of nightmares compared to prospective measures like logs and diaries. However, Zunker et al. [37] showed that the difference between these two approaches measuring nightmare frequency was relatively small (effect size = 0.101), that is, the type of measurement should have only minor effects on the findings. The participants reported the name of their home town but were not asked about additional information like whether they live in the city center or in the suburbs. As chronotype in urban settings might be shifted slightly to eveningness by outdoor light [32,33], this information would have been helpful.

Although there was a negative bivariate correlation between chronotype and nightmare frequency within this data set, morningness was associated with fewer nightmares [18]—a finding which is in line with previous studies [16,17], including age, gender, education, place of residence size, and personality variables, especially neuroticism, indicating there is no direct relationship between nightmares and chronotype. The relationship seems to be mediated by neuroticism that is higher in evening types [14]. We also did not find an association between chronotype and nightmare distress. Therefore, more studies including measures of psychopathology—as this is a main factor associated with nightmare frequency and nightmare distress [9]—are needed to clarify the question as to whether chronotype might contribute independently to nightmares.

Interestingly, we found a small but significant association between morningness and dream recall frequency—if nightmare frequency was partialed out. One might speculate—as dream recall frequency is also associated with conscientiousness in this sample—whether regular sleep patterns, more often found in morning types than in evening types who can show dramatic social jet lags on weekends with very late bedtimes [30], might facilitate dream recall due to less pronounced sleep inertia [38]. However, empirical evidence for this hypothesis has not yet been provided.

In contrast to previous findings [25,26], we did not find a relationship between urbanicity and dream recall frequency. Moreover, living in an urban setting was also not related to heightened nightmare frequency, a finding which is in line with Schredl [23] but not with Schredl [21], who reported a small but significant relationship between nightmares frequency and place of residence size. For a more detailed analysis, it would be helpful to elicit more information about the urban area the individuals live in, e.g., city center vs. suburbs. This information was not available in the present data; Sandman et al. [24] reported no differences regarding nightmare frequency for persons living in inner cities or in city outskirts. Keep in mind that education, which is correlated with monthly income, was controlled in the analysis, but this might be only a proxy for the kind of neighborhood the participants live in. Interesting and not expected was the finding that persons in rural areas experience more nightmare distress than persons in urban settings—even though the effect was quite small. One possible explanation might be a factor studied by Schredl et al. [39]: beliefs about nightmares. Beliefs like “Nightmares predict the future” and “Nightmares contain clues to unconscious fears.” are associated with increased nightmare distress—independently from nightmare frequency. As these beliefs are related to lower educational levels [39] and nightmare distress was also higher in persons with lower educational levels in the present sample, one might speculate as to whether individuals living in urban settings have more access to more up-to-date models of nightmare etiology (nightmares are related to everyday stress levels, in addition to disposition). This is an interesting hypothesis that could be studied in the future, e.g., testing the effect of psychoeducation about nightmare etiology on nightmare distress. It is also of clinical relevance since disseminating accurate information about nightmares might help to reduce nightmare distress in individuals suffering from nightmares.

To summarize, chronotype and urbanicity might be associated with nightmares and dream recall but research findings so far are contradictory. Future studies should include more detailed data on psychopathology in general, nightmare beliefs, participants’ neighborhoods, etc., in order to clarify whether these two variables that are associated with mental disorders [15,20] contribute independently to nightmare frequency and nightmare distress.

## 4. Method

### 4.1. Participants

Overall, 2492 persons (1437 women, 1055 men) with a mean age of 47.75 ± 14.41 years (range: 17 to 93 years) participated in the study. Concerning educational level, 0.8% (*n* = 20) had no degree, 10.47% (*n* = 261) had 9 years of schooling, 28.33% (*n* = 706) had O-levels (about 10 years), 26.00% (*n* = 648) had A-levels (“Abitur”), 31.7% (*n* = 790) obtained a University degree, and 2.69% (*n* = 67) held a doctorate. The sizes of the places of residence were distributed as follows: 12.97% (*n* = 323) lived in towns with less than 5000 inhabitants, 8.23% (*n* = 205) with 5000 to 9999 inhabitants, 10.76% (*n* = 268) with 10,000 to 19,999 inhabitants, 13.73% (*n* = 312) with 20,000 to 49,999 inhabitants, 7.59% (*n* = 189) with 50,000 to 99,999 inhabitants, 26.63% (*n* = 663) with 100,000 to 499,999 inhabitants, and 20.08% (*n* = 500) lived in cities with 500,000 or more inhabitants.

### 4.2. Research Instruments

#### 4.2.1. Dream Questionnaire

For eliciting dream recall frequency, a 7-point scale (coded as 0 = never, 1 = less than once a month, 2 = about once a month, 3 = about 2 to 3 times a month, 4 = about once a week, 5 = several times a week, 6 = almost every morning) was presented. The retest reliability of this scale is high: r = 0.85 for an average interval of about 55 days [40]. To assess nightmare frequency, an 8-point rating scale (“How often do you experience nightmares?” 0: never, 1: less than once a year, 2: about once a year, 3: about 2 to 4 times a year, 4: about once a month, 5: about 2 to 3 times a month, 6: about once a week, 7: several times a week) with the following definition was presented: “Nightmares are dreams with strong negative emotions that result in awakening from the dreams. The dream plot can be recalled very vividly upon awakening.” [41] Retest reliability of the nightmare frequency scale was high [42]: r = 0.75 (four weeks retest interval). Nightmare distress was measured with a five-point scale “If you currently experience nightmares, how distressing are the nightmares?” (0 = Not at all distressing, 1 = Not that distressing, 2 = Somewhat distressing, 3 = Quite distressing, and 4 = Very distressing). Retest reliability for a two-week interval was somewhat lower r = 0.673 [41].

#### 4.2.2. Chronotype Questionnaire

For measuring chronotype, four items of the German version of the Morning–Evening Questionnaire (MEQ) [31], a translation of the original publication by Horne and Ostberg [43], were used: “Considering only your own “feeling best” rhythm, at what time would you get up if you were entirely free to plan your day? (the presented scale ranged from 5 a.m. to 1 p.m.)”, “Considering only your own “feeling best” rhythm, at what time would you go to bed if you were entirely free to plan your evening?” (the presented scale ranged from 8 p.m. to 4 a.m.), “You wish to be at your peak performance for a test which you know is going to be mentally exhausting and lasting for two hours. You are entirely free to plan your day and considering only your own “feeling best” rhythm which ONE of the four testing times would you choose?” (8 to 10 a.m., 11 a.m. to 1 p.m., 3 to 5 p.m., and 7 to 9 p.m.), and, finally, “One hears about “morning” and “evening” types of individuals. Which ONE of these types do you consider yourself to be?” (definitely evening type, more evening than morning type, more morning than evening type, definitely morning type). The chronotype sum score ranged from 2 (extreme eveningness) to 22 (extreme morningness) (for the exact scoring rules, see Griefahn, Künemund, Bröde, and Mehnert [31]). The internal consistency (Cronbach’s alpha) of the four-item scale was high (Table 4). The four-item scale correlated at r = 0.835 with the full MEQ in a sample of 71 students [18].

#### 4.2.3. Personality Questionnaire

The Big Five personality factors were measured with the German version of the NEO-FFI-30 that includes 30 items [44]. For each of the five factors (neuroticism, extraversion, openness to experience, agreeableness, and conscientiousness), the sum score of the six corresponding items was computed. The internal consistencies (Cronbach’s alpha) for the current sample are depicted in Table 4. These figures were comparable to those of the 60-item version of the NEO-FFI ranging from r = 0.67 (openness to experience) to r = 0.81 (neuroticism) [45].

### 4.3. Procedure

The participants completed the online survey (language: German) between 23 March 2015 and 8 April 2015. Within the online panel [46], persons with an interest in online studies and with heterogenic demographic backgrounds were registered. The link for the study was sent to all panelists (about 10,000). For some surveys, prizes or money are offered for study participation, but this study was completely voluntary and unpaid. The participants registered in the panel provided their postal code and city name, and current place of residence sizes were obtained via internet. A total of 2421 participants lived in Germany, 44 in Austria, and 18 in Switzerland. A total of 9 German citizens lived in other countries.

Statistical procedures were carried out with the SAS software package (Version 9.4, SAS Institute, Cary, North Carolina, NC, USA) for Windows. We carried out a parametric regression to assess the influence of age, gender, education, place of residence size, and personality on chronotype. Ordinal regressions (cumulative logit analyses) were used for analyzing the effect of different predictors on dream recall frequency, nightmare frequency, and nightmare distress. The SAS “Logistic” procedure provides an adjusted pseudo-R^2^ according to Nagelkerke which is roughly comparable to R^2^ in parametric regressions.

## Figures and Tables

**Table 1:** Dream recall frequency (*n* = 2492).

Category	Frequency	Percent
Almost every morning	211	8.47%
Several times a week	638	25.60%
About once a week	498	19.98%
About 2 to 3 times a month	363	14.57%
About once a month	225	9.03%
Less than once a month	379	15.21%
Never	178	7.14%

**Table 2:** Nightmare frequency (*n* = 2492).

Category	Frequency	Percent
Several times a week	88	3.53%
About once a week	128	5.14%
About 2 to 3 times a month	235	9.43%
About once a month	313	12.56%
About 2 to 4 times a year	512	20.55%
About once a year	296	11.88%
Less than once a year	436	17.50%
Never	484	19.42%

**Table 3:** Nightmare distress (*n* = 2008).

Category	Frequency	Percent
Very distressing	95	4.73%
Quite distressing	325	16.19%
Somewhat distressing	658	32.77%
Not that distressing	610	30.38%
Not at all distressing	320	15.94%

**Table 4:** Mean and standard deviations of chronotype and personality variables.

Variable	Mean ± SD	*n*	Cronbach’s Alpha
Chronotype	13.56 ± 4.38	2492	0.771
Neuroticism	1.48 ± 0.91	2490	0.883
Extraversion	2.10 ± 0.65	2488	0.761
Openness to experience	2.46 ± 0.74	2488	0.750
Agreeableness	2.86 ± 0.66	2489	0.751
Conscientiousness	2.95 ± 0.62	2492	0.798

**Table 5:** Linear regression for chronotype score (*n* = 2479).

Variable	Chronotype Scale		
	SE	t	*p*
Age	0.1001	4.8	<0.0001
Gender	0.0017	0.1	0.9353
Education	0.0452	2.2	0.0253
Place of residence size	−0.0588	−3.0	0.0030
Neuroticism	0.0311	1.3	0.1922
Extraversion	0.0488	2.2	0.0267
Openness to experience	−0.1012	−5.0	<0.0001
Agreeableness	0.0029	0.1	0.8915
Conscientiousness	0.1461	6.6	<0.0001
Model fit	R^2^ = 0.0459, F = 14.2, *p* < 0.0001		

SE = standardized estimates.

**Table 6:** Ordinal regressions for dream recall frequency (*n* = 2479).

Variable	Analysis 1			Analysis 2		
	SE	χ^2^	*p*	SE	χ^2^	*p*
Age	−0.1037	24.6	<0.0001	−0.0245	1.3	0.2510
Gender	0.0222	1.1	0.2855	0.0199	0.9	0.3440
Education	0.0311	2.4	0.1234	0.0184	0.8	0.3674
Place of residence size	−0.0041	0.0	0.8372	−0.0127	0.4	0.5240
Neuroticism	0.1855	59.4	<0.0001	0.0028	0.0	0.9112
Extraversion	−0.0047	0.0	0.8301	0.0065	0.1	0.7710
Openness to experience	0.2041	95.9	<0.0001	0.1552	54.2	<0.0001
Agreeableness	0.0340	2.5	0.1150	0.0470	4.7	0.0307
Conscientiousness	0.1125	25.5	<0.0001	0.1000	19.7	<0.0001
Chronotype	−0.0319	2.5	0.1120	−0.0455	5.0	0.0252
Nightmare frequency				0.5802	558.4	<0.0001
Model fit	R^2^ = 0.0929, χ^2^ = 230.0, *p* < 0.0001			R^2^ = 0.2901, χ^2^ = 395.3.2, *p* < 0.0001		

SE = standardized estimates.

**Table 7:** Ordinal regressions for nightmare frequency and nightmare distress.

Variable	Nightmare Frequency (*n* = 2479)			Nightmare Distress (*n* = 1997)		
	SE	χ^2^	*p*	SE	χ^2^	*p*
Age	−0.1853	77.1	<0.0001	0.0657	7.3	0.0068
Gender	0.0225	1.2	0.2817	0.1194	23.9	<0.0001
Education	0.0416	4.2	0.0396	−0.0934	15.9	<0.0001
Place of residence size	0.0219	1.2	0.2690	−0.0597	6.7	0.0095
Neuroticism	0.3958	256.3	<0.0001	0.3349	133.1	<0.0001
Extraversion	−0.0199	0.8	0.3672	0.0300	1.4	0.2378
Openness to experience	0.1381	44.4	<0.0001	0.0908	14.4	<0.0001
Agreeableness	−0.0108	0.3	0.6153	0.0116	0.2	0.6418
Conscientiousness	0.0541	5.9	0.0150	−0.0101	0.2	0.6921
Chronotype	0.0324	2.6	0.1073	−0.0293	1.6	0.2089
Nightmare frequency				0.4181	261.0	<0.0001
Model fit	R^2^ = 0.2011, χ^2^ = 315.1, *p* < 0.0001			R^2^ = 0.3031, χ^2^ = 594.5, *p* < 0.0001		

SE = standardized estimates.
